# Novel User-Friendly Application for MRI Segmentation of Brain Resection following Epilepsy Surgery

**DOI:** 10.3390/diagnostics12041017

**Published:** 2022-04-18

**Authors:** Roberto Billardello, Georgios Ntolkeras, Assia Chericoni, Joseph R. Madsen, Christos Papadelis, Phillip L. Pearl, Patricia Ellen Grant, Fabrizio Taffoni, Eleonora Tamilia

**Affiliations:** 1 Fetal Neonatal Neuroimaging and Developmental Science Center (FNNDSC), Newborn Medicine Division, Department of Pediatrics, Boston Children’s Hospital, Boston, MA 02115, USA; georgios.ntolkeras@childrens.harvard.edu (G.N.); assia.chericoni@gmail.com (A.C.); ellen.grant@childrens.harvard.edu (P.E.G.); 2 Advanced Robotics and Human-Centered Technologies-CREO Lab, Università Campus Bio-Medico di Roma, 00128 Rome, Italy; f.taffoni@unicampus.it; 3 Baystate Children’s Hospital, Springfield, MA 01199, USA; 4 Epilepsy Surgery Program, Department of Neurosurgery, Boston Children’s Hospital, Harvard Medical School, Boston, MA 02115, USA; joseph.madsen@childrens.harvard.edu; 5 Jane and John Justin Neurosciences Center, Cook Children’s Health Care System, Fort Worth, TX 76104, USA; christos.papadelis@cookchildrens.org; 6 Division of Epilepsy and Clinical Neurophysiology, Boston Children’s Hospital, Harvard Medical School, Boston, MA 02115, USA; phillip.pearl@childrens.harvard.edu

**Keywords:** brain resection, epilepsy surgery, MRI, region growing, image segmentation

## Abstract

Delineation of resected brain cavities on magnetic resonance images (MRIs) of epilepsy surgery patients is essential for neuroimaging/neurophysiology studies investigating biomarkers of the epileptogenic zone. The gold standard to delineate the resection on MRI remains manual slice-by-slice tracing by experts. Here, we proposed and validated a semiautomated MRI segmentation pipeline, generating an accurate model of the resection and its anatomical labeling, and developed a graphical user interface (GUI) for user-friendly usage. We retrieved pre- and postoperative MRIs from 35 patients who had focal epilepsy surgery, implemented a region-growing algorithm to delineate the resection on postoperative MRIs and tested its performance while varying different tuning parameters. Similarity between our output and hand-drawn gold standards was evaluated via dice similarity coefficient (DSC; range: 0–1). Additionally, the best segmentation pipeline was trained to provide an automated anatomical report of the resection (based on presurgical brain atlas). We found that the best-performing set of parameters presented DSC of 0.83 (0.72–0.85), high robustness to seed-selection variability and anatomical accuracy of 90% to the clinical postoperative MRI report. We presented a novel user-friendly open-source GUI that implements a semiautomated segmentation pipeline specifically optimized to generate resection models and their anatomical reports from epilepsy surgery patients, while minimizing user interaction.

## 1. Introduction

### 1.1. Epilepsy Surgery and Resected Brain Cavity

Brain surgery is the best available treatment for patients suffering from drug-resistant epilepsy (DRE) [1] who represent between 20–30% of all the epilepsy cases [2,3,4]. The objective of resective epilepsy surgery is the complete resection or disconnection of the epileptogenic zone, which is defined as “the area of cortex indispensable for the generation of clinical seizures” [5]. Delineating the epileptogenic zone, therefore, is key to the success of epilepsy surgery; yet, this zone is a fully theoretical concept: no diagnostic modality, that is currently available, can measure the entire epileptogenic zone directly. Prior to the surgery, we cannot exclude the presence of a potential epileptogenic zone that would only become clinically apparent postoperatively (once the patient continues to have epileptic seizures, coming for a spared part of the brain). The more recent concept of the epileptogenic zone as “the minimum amount of cortex that must be surgically resected (or completely disconnected) to produce seizure freedom” apparently provides a more objective definition of this zone [6]. This provides an objective and practical criterion for the testing of this theoretical concept. It means that seizure-freedom after surgery is evidence that the whole epileptogenic zone was included in the resected area [5]. Therefore, delineating the exact margins of the resected brain tissue epileptogenic zone postoperatively is a key step to study the epileptogenic zone and understand what characterizes it.

In patients with DRE, neuroimaging techniques, and particularly magnetic resonance imaging (MRI) coupled with electroencephalography (EEG) or magnetoencephalography (MEG) data, represent the techniques of choice to carefully plan the intervention and evaluate its effectiveness [4,7,8]. Many research efforts are dedicated to extricating the associations between specific characteristics of the resected brain tissue and patients’ postsurgical outcome [2,9,10,11,12,13,14]: identifying which properties of the resected tissue (also relative to the non-resected tissue) are associated with postsurgical seizure freedom constitutes the first step towards the identification and validation of epilepsy biomarkers or outcome predictors in epilepsy surgery. Recent studies on DRE have showed how the presence of specific biomarkers–such as EEG or MEG high-frequency oscillations [2,10,15,16,17,18], EEG phase-amplitude coupling [19], MEG discharges [9,20,21,22], multi-feature intracranial EEG patterns [23,24]–in the resected tissue, relative to that in non-resected tissue, is associated with high probability of good outcome.

Therefore, the accurate segmentation of the resected brain cavity in neuroimaging data represents a crucial step for testing the epileptogenic zone concept as well as improving epilepsy surgery outcome prediction that is key for clinical decision-making.

### 1.2. Technical Aspects, Cons and Pros of Existing Methods

A wealth of different algorithms for automated, semiautomated and interactive segmentation of brain structures or abnormalities (such as tumors or lesions) have been developed in the last 20 years [7,25,26,27,28,29,30], but none of them have been specifically designed to investigate neurosurgical resections [31]. This task is typically performed manually through a slice-by-slice tracing of MR images [7,8,26,31], which is a time-consuming procedure, requires an expert and is still prone to inter- and intra-rater errors.

An ideal medical image-segmentation process of a specific region of interest (ROI; e.g., a generic anatomical structure, a tumor, or in our case, a resection cavity) is characterized by: (i) minimum user interaction, (ii) fast computation, (iii) accurate and robust results [28], (iv) quantitative data on different sizes, shapes and location [8]. Only one study, to the best of our knowledge, investigated the accuracy of different segmentation methods for resected brain areas [31]: In this study, Gau et al. showed how the semiautomated (boundary-based) segmentation of the ITK-SNAP software [8] outperformed the fully automated (statistical-based) approach of the lesion_GNB software [32]. Statistical-based fully automated methods rely on parametric models of intensity distribution, usually available for the healthy brain: these algorithms delineate the brain regions by tuning such parameters and effectively address the problem of segmentation when intensity distributions of different tissues are a priori known [26]; yet in real cases, especially for pathological brains, no prior knowledge about the data distribution is available [28]. On the other hand, the semiautomated segmentation method used by Gau et al. [31] was not specifically developed to delineate resection cavities and requires substantial inputs by the user, who must manually initialize the starting contour in several layers of the image, which can be both inaccurate and tedious, and could lead to errors caused by local gradients [27,28]. This is due to the fact that boundary-based segmentation methods [8] use local image features (i.e., edges) to attract an evolving contour delineating the ROI onto the target edges. The most common limitations of this class of algorithms are the long computation time, and the necessity to manually initialize the starting contour. Therefore, there is an evident need for a semiautomated user-friendly tool to accurately delineate resection cavities on patients’ MRIs. This tool should be conceived to be used to any nonexpert user without requiring fine tuning of multiple unintuitive parameters as in the currently available systems. While the specific segmentation of surgical resections has not been largely investigated yet, several semiautomated approaches have been developed for other medical applications, such as tumors, vessels or lesion segmentation [33,34,35]. Semiautomatic approaches have the advantage of combining the efficiency and repeatability of automatic segmentation with the sound judgement that can only come from human expertise, resulting in higher overlaps between the outcome of the algorithm and the human segmentation compared to fully automated approaches (0.78 vs. 0.58 [31]). Among this type of approaches, region-growing-based methods have been largely explored [27,36,37,38]. These methods provide a simpler way to delineate a ROI on an image, since they require the user to manually select only one seed (whose value falls in an interval that is representative for the segmentation task), which then typically grows (growing region) until the difference between its intensity and that of the next candidate voxels is higher than a predefined threshold. Region-growing methods can be successfully applied to segment ROIs with homogeneous intensity, in which the seed placement is relatively easy, as in the case of neurosurgical resections (whose intensity is homogenous and markedly different from the surrounding voxels). Lack of homogeneity may otherwise produce inaccurate segmentations that leak outside the ROI; besides this, if the ROI has an intensity level that is similar to other regions or to the background, the growing process may leak into them as well [27]. When delineating a surgical resection in the brain, while the lack of homogeneity does not represent an issue, the possible leakage into the MRI background does.

### 1.3. Our Contribution

Based on all these considerations and on the results from previous neuroimaging studies, in the present work we aimed at developing a semiautomated segmentation pipeline to delineate brain resections on the MRI of patients who had epilepsy surgery. We used a hybrid approach that integrates a region-growing process on the patient’s postoperative MRI with the definition of a patient-specific mask through preoperative MRI segmentation in order to overcome leakage into the background. We tested a variety of settings for image processing and region growth and identified the most appropriate configuration to optimize the output, thus reducing the considerable amount of human expertise otherwise needed for accurate tuning. Finally, we developed a semiautomated, user-friendly graphical user interface (GUI) for brain MRI segmentation, specifically tuned and optimized to generate an accurate model of the resection and its neuroanatomical labeling, while requiring minimal user interaction and image-processing expertise. The GUI source code is freely available as a MATLAB-based application (https://github.com/rbillardello/BrainResectionApp (accessed on 14 April 2022)).

## 2. Materials and Methods

Data from 35 patients who underwent focal epilepsy surgery at the Epilepsy Center of Boston Children’s Hospital (Boston, MA, USA) were included. Patients who had hemispherectomy, corpus callosotomy or thermal ablation were excluded. For each patient, we retrieved the pre- and postoperative MRIs (T1-weighted volumetric MRI), which had been acquired as part of their routine pre- and postsurgical evaluation.

The segmentation algorithm we propose here integrates a region-growing approach, which grows on the postoperative MRI, and the definition of a patient-specific mask through the preoperative MRI segmentation. Thus, it requires the following inputs: (1) postoperative MRI (where the resection cavity is present); (2) patient-specific preoperative brain mask (to define the initial brain space), and (3) the location of one initial seed within the resection cavity (to initiate the region-growing process).

### 2.1. Preprocessing of Input Images

Preoperative and postoperative MRI were co-registered in Brainstorm [39] using the statistical parametric mapping (SPM12) toolbox with reslice [40]. Postoperative MR images were then exported from Brainstorm to MATLAB (The MathWorks Inc., Natick, MA, USA) and converted to uint8 type. We performed the FreeSurfer [41] cortical reconstruction process (recon-all) on the preoperative MRIs to extract each patient’s brain mask (skull stripping) and also obtain a cortical parcellation (based on the Desikan–Killiany atlas) [42]. The brain mask was needed to limit the region-growing to the brain space (see Section 2.2), while cortical parcellation was needed to provide a neuroanatomical report of the resection (see Section 2.5).

To identify the optimal segmentation pipeline, we assessed the performance of the region-growing output when using different standardization methods as well as when setting threshold or smoothing parameters to different possible values. Regarding the standardization process used to treat the input image (postoperative MRI), we chose to use and test two built-in MATLAB normalization methods for image contrast, which remap the intensity distribution according to the image class range: the linear contrast stretch adjustment (ADJ) and the max-min normalization (NORM). The ADJ linearly remaps the intensity distribution of the image over the limits derived by saturating the top and bottom voxel values from the whole MRI. The NORM remaps the image intensity according to the intensity span (max-min) measured in the whole MRI. In addition, we also decided to implement the ”local” version of these two methods (meaning that the normalization was performed slice by slice rather than by the whole image) since this would favor the region-growing process to grow intra-slice before moving to the next. Thus, we ended up testing the following four methods: (1) Global ADJ (ADJ-G); (2) Local ADJ (ADJ-L); (3) Global NORM (NORM-G); (4) Local NORM (NORM-L).

**Figure 1:**
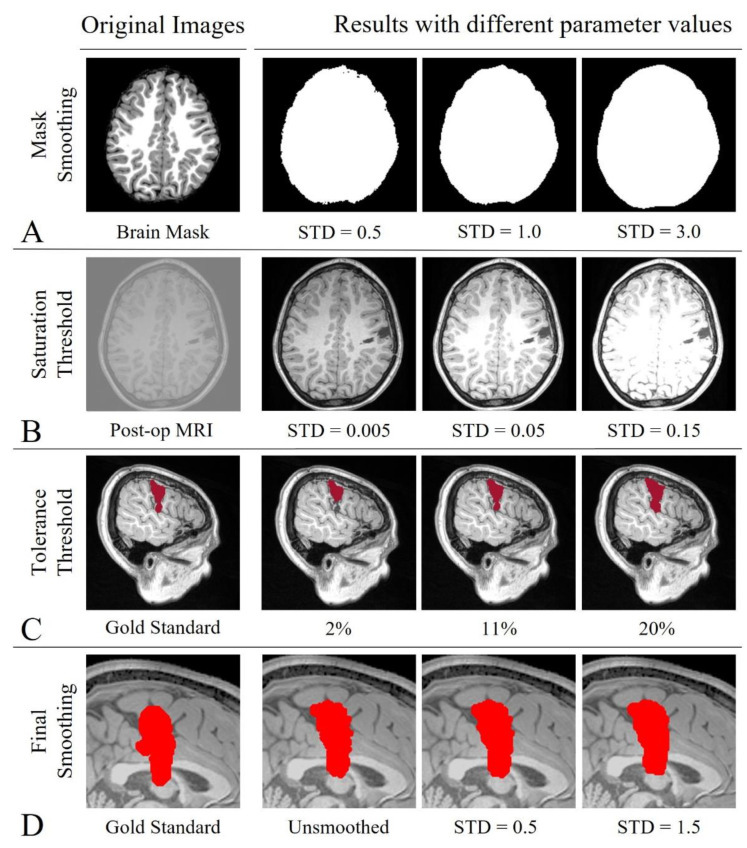
Results from low (2nd column) to high (4th column) parameter values. Mask Smoothing (**A**-1st row): original preoperative MRI brain mask (left) and three different binarized smoothed masks (right); Saturation Threshold (**B**-2nd row): low-contrast original postoperative MRI (left) and three contrast-adjusted images (right); Tolerance Threshold (**C**-3rd row): manual traced resection (left) and three examples of resections obtained with increasing tolerance value (right); and Final Smoothing (**D**-4th row): manual traced resection (left) and three different values of smoothing.

We varied the values of three or four threshold parameters (when using NORM or ADJ, respectively) to identify the best-performing set of values, so that we could design an optimized segmentation pipeline that reduces the user interaction and need for tuning. Table 1 reports details about the parameters and their values that were tested to optimize the segmentation pipeline depending on the standardization process (ADJ and NORM, whether global or local). Section 2.3 describe how we tested the performance of all the possible segmentation pipelines. Figure 1 shows a schematic of how the parameters’ tuning modifies the inputs or output of the segmentation pipeline. To preprocess the patient’s brain mask, we used a 3D Gaussian smoothing kernel filter to smooth its margins and then we binarized the output to again obtain a logical brain mask (including all the voxels with values > 0.01) where to confine the region-growing algorithm: Figure 1A shows how we varied the values of standard deviation (STD) for the brain-mask smoothing, starting from low (STD = 0.5, resulting in a more conservative brain mask) to higher values (STD = 3, resulting in a more extended and smoothed brain mask). To preprocess the postoperative MRI using the ADJ method, we varied the saturation threshold (Figure 1B). We also used the preoperative cortical-surface-parcellation file from Freesurfer to obtain a logical map of the brain ventricles and exclude them from the logical brain mask (although this only improves the algorithm performance if the resection is in the proximity of the ventricle space; it is not needed otherwise).

### 2.2. Region-Growing Algorithm

The proposed segmentation algorithm requires the placement of a seed within the resected cavity on the postoperative MRI by the human user. Such a user-based initialization was thus performed by a human user who was instructed to manually place the initial seed for each patient based on the following instructions: (i) the initial seed must be in the center of the cavity rather than on the border; (ii) the initial seed must be one of the darkest voxels in the cavity (i.e., have low intensity values). Different slices and views were presented to the user, who was left free to select any point. The user was asked to select a new seed whenever he chose a point outside the gold standard or an outlier with a high intensity value (where “high” means above the mean intensity value of the gold standard).

Starting from the initial seed, neighbor voxels (6-Neighboring) that are in the brain map were added to a “neighbors list”. Then, the difference between the intensity value of each element in this list and the mean intensity of voxels in the evolving resection region was calculated. If the minimum difference was below a tolerance threshold (see Table 1 for the definition of this parameter), the corresponding voxel was moved from the neighbors list to the growing region, and its neighbor voxels were then analyzed. The region-growing algorithm stopped when there were no more neighbor voxels or the minimum intensity difference between the growing region and the neighbors list was higher than the tolerance threshold. As Table 1 reports, seven values of tolerance threshold were tested. Finally, the outcome of the region-growing algorithm was smoothed using a 3D Gaussian smoothing kernel filter, for which we tested and compared four different conditions (see Table 1). Results were then again binarized (threshold equal to 10% of the maximum value) to have a final logical map of the resection. Figure 1C,D show how the output changes when tuning tolerance and final smoothing. Section 2.4 will also present how we simulated different seed placements and compared their performance.

### 2.3. Performance Assessment

The gold standard we used to assess the performance of our segmentation was defined through manual delineation of the resection cavity on the coregistered MRIs; this was performed using Slicer 4.11 software by an experienced rater, who was blind to the results of our algorithm. The segmentation outcome was compared with the hand-drawn gold standard by calculating the dice similarity coefficient (DSC) [43]. DSC is an efficient and robust metric of both overlap and reproducibility of MRI segmentation outputs [7,8,25,26,31], calculated as:

(1) $$DSC=2\times \frac{\left|X\cap^{\;}Y\right|}{\left|X\right|+\left|Y\right|}$$

where *X* and *Y* are the sets of voxels labeled as resected based on the gold standard and the algorithm, respectively; “∩” represents the intersection of two sets, and the vertical bars represent their cardinality (i.e., number of elements). *DSC* values above 0.6 are considered as “good”, above 0.7 as “high”, and above 0.8 as “excellent” [31].

For each combination of parameters, we calculated the mean DSC across our cohort and compared the results to identify the best sets of parameters. More precisely, for each parameter in Table 1, we assessed whether and how the DSC changed when varying the value of that parameter only (while keeping the others fixed): for each parameter, Wilcoxon signed rank test was used between each pair of consecutive values to identify the optimal value as the one maximizing the DSC, i.e., showing DSC significantly higher than the others. Wilcoxon signed-rank test was also used to compare performance between optimized pipelines within our cohort. Results are reported as median (inter-quartile range, IQR); *p*-values below 0.05 were considered significant and Bonferroni correction was applied for multiple comparisons.

By setting each parameter to its optimal value, we defined our optimized segmentation pipeline, tested its inter-rater reliability and used it to generate the automated anatomical report of the resection as described in the following sections. Furthermore, when we developed the GUI, we chose to leave the user the possibility to tune only one parameter, while all the others were set to their optimal value with no possible user interaction. The optimal values for each parameter were defined based on the results of the statistical comparisons described above, which pointed out at the values maximizing the DSC in our cohort. We gave the user the option to tune one parameter in order to take advantage of human judgment and reasoning; thus, we left the tolerance threshold available for tuning because of its intuitiveness: higher tolerances lead to more inclusiveness and thus to larger resection volumes, while lower tolerances lead to less inclusiveness and thus smaller resection volumes. To test if this setting allows a good performance to be reached, we computed the maximum possible DSC that is obtainable by only leaving the tolerance threshold to change (maximum selectable, MaxS, among seven options), with the maximum possible (MaxP) DSC for each patient, which is obtainable by leaving all the parameters to change (among 504 options). Then, we calculated a mean quadratic discard (MQD) between the two:

(2) $$MQD=\sqrt{\frac{\sum_{i=1}^{N}{\left(MaxSi-MaxPi\right)}^{2}}{N}}$$

where *MaxSi* and *MaxPi* are the maximum selectable and maximum possible DSC for the ith patient, and N is the number of patients.

We finally tested whether performance (*MaxS*) depended on factors such as resection volume or patient’s age through correlation (Pearson) analysis, or temporal versus extratemporal cases through Wilcoxon rank-sum test.

### 2.4. Inter-Rater Reliability

**Figure 2:**
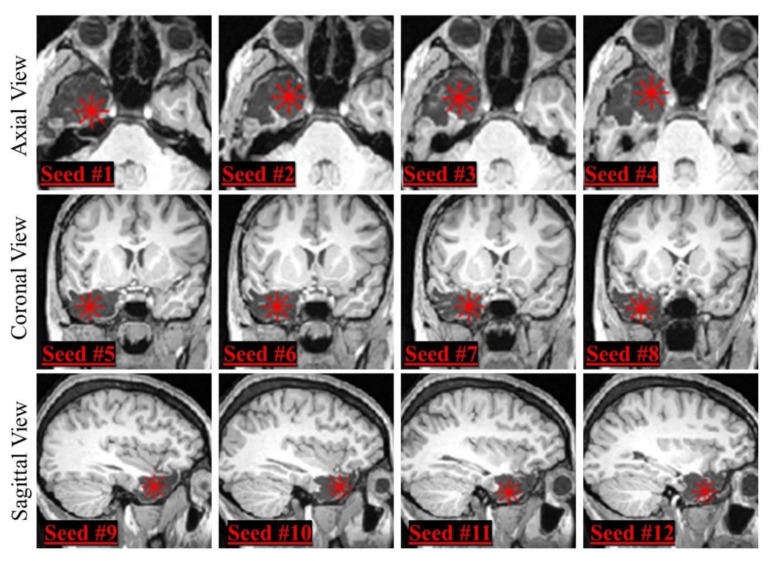
Seed positioning in different MRI views. We selected four seeds (indicated by red stars) from different slices from each MRI view (first row: axial view, second row: coronal view, third row: sagittal view), obtaining a total of 12 different seeds for each patient.

To simulate the manual placement of the seed by different users, a human user hand-picked 12 seeds per patient (i.e., 11 additional seeds in the proximity of the initial selected seed), chosen from different MRI views and slices as Figure 2 shows: the user was asked to place four seeds per view on slices that were two voxels apart. Since patients could have thin resection cavities in one of the three directions, we chose to place the multiple, proximal seeds no more than 2 voxels apart in all subjects (meaning that seeds were distributed over a span of 6 voxels per direction); this ensured that we could be consistent across our cohort, including subjects with small resections. Then, the optimized segmentation pipelines were used to create a resection model from each of the 12 seeds per patient, and we calculated inter-rater reliability via two metrics: DSC and volume ratio (ratio of number of voxels) between the segmentation outputs obtained from different seeds. We only tested initial seeds placed inside the resection cavity, and this is a prerequisite of the proposed segmentation tool: the semiautomated tool we propose is meant to be used to facilitate the human reader, who is able to identify the resection, in delineating the whole cavity (its volume and margins) accurately and rapidly.

### 2.5. Automated Anatomical Report

**Figure 3:**
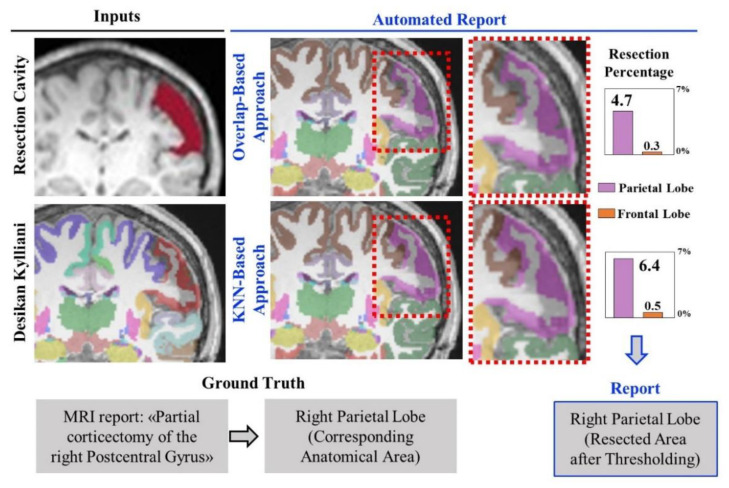
Automated generation of the resection anatomical report. Example of inputs (right: segmentation of the resection cavity, preoperative Desikan–Killiany cortical parcellation) and outputs (left: resection percentages of each anatomical area, and list of resected areas). Each cortical area of the Desikan–Killiany is associated with one anatomical area (frontal, temporal, parietal, occipital, cingulate, hippocampus, amygdala). Two approaches (overlap- and KNN-based) were used to estimate the resection percentage of each anatomical area and then generate an anatomical report, which was then validated by comparison with the ground truth (clinical MRI postsurgical report). In this example, both automated reports indicate right parietal lobe resection, consistently with the ground truth.

To generate an anatomical report of the resection, we developed and tested two approaches based on the comparison between each patient’s resection model and their Desikan–Killiany atlas parcellation (obtained via Freesurfer). The parcellation file contains a 3D image, where each voxel has an anatomical label that identifies a sublobe to which it belongs, and to whom we assigned an anatomical area [44] among the following: frontal, temporal, parietal, occipital, cingulate, hippocampus, amygdala. Figure 3 shows the brain parcellation file for one of our patients and the automated report generated based on the two different approaches that we implemented to assign the resection voxels to an anatomical area. In the first approach, we coregistered the resection model with the preoperative parcellation file and assessed its overlap with all the different anatomical areas (overlap-based approach). Because of the lack of exact alignment between preoperative and postoperative brain margins and gyrification, many voxels within the postoperative resection model may erroneously overlap with an unknown label in the preoperative parcellation file (corresponding to background or cerebrospinal fluid, CSF). For this reason, we implemented a second approach to assign all the unknown voxels within the segmented resection based on their neighbor voxels: we used the patient-specific preoperative parcellation labels—different from “unknown”—to train a k-nearest-neighbor (KNN) classifier, which was then used to label all the unknown voxels of the resection (meaning assigning them to the nearest anatomical area).

To assess the accuracy of the two approaches, we compared them with the ground truth that consisted of the anatomical areas described as surgically resected in the postoperative MRI clinical report. Thus, we calculated the resection percentage of each preoperative anatomical area (as the number of resection voxels assigned to an area divided by the total number of the area’s voxels) and classified an area as “resected” if showing a percentage above a minimum threshold, or “nonresected” otherwise. Since the optimal threshold to classify an area as resected was not known a priori, we varied the thresholds from 0% (where all lobes with at least one voxel in the resection model are considered as “resected”) to 100% (where all lobes are considered as “nonresected”). For each threshold, we assessed true or false positives (resected areas present or not in the patient’s clinical report), and true or false negatives (nonresected areas absent or not from the patient’s clinical report). Based on this, the automated anatomical report of the resection of each patient was regarded as:

- Fully concordant: complete match between the resected areas of the automated and clinical report (no false classifications).
- Partly concordant, including three scenarios: (i) the automated report points to all the areas of the clinical report plus additional false-positive areas (clinical report is a subset of the automated); (ii) automated report points to only some of the areas of the clinical report, thus there are false negatives (automated report is a subset of the clinical); or (iii) automated report points to only some of the areas of the clinical report (there are false negatives) plus additional false positives.
- Discordant: no match between any of the areas reported in the automated and clinical report (no true classifications).

Furthermore, for each threshold, we estimated accuracy, sensitivity and specificity across our entire cohort, and a leave-one-out cross-validation (LOOCV) was performed to find the optimal threshold on the resection percentage (to classify an area as “resected”) based on three different criteria: (i) maximizing the number of fully concordant reports in our cohort while minimizing discordances; (ii) maximizing the overall accuracy of our outputs; or (iii) optimizing the receiver operating characteristic (ROC) curve (optimal operating point).

## 3. Results

Our cohort included 35 patients (11 females) with a median age at surgery of 12 years (8.25–16.75); 16 of them had temporal resections (vs. 19 extratemporal) and 74% of them (*n* = 26) became seizure-free (Engel scale 1) after resective epilepsy surgery. The average volume of resected brain tissue per patient (based on the gold-standard manual definition) was 18.89 (13.20–36.74) cm^3^ without difference (*p* = 0.75) between good (Engel 1; 17.7 (13.8–36.9) cm^3^) and poor outcomes (Engel > 1; 31.6 (9.6–38.4) cm^3^).

**Figure 4:**
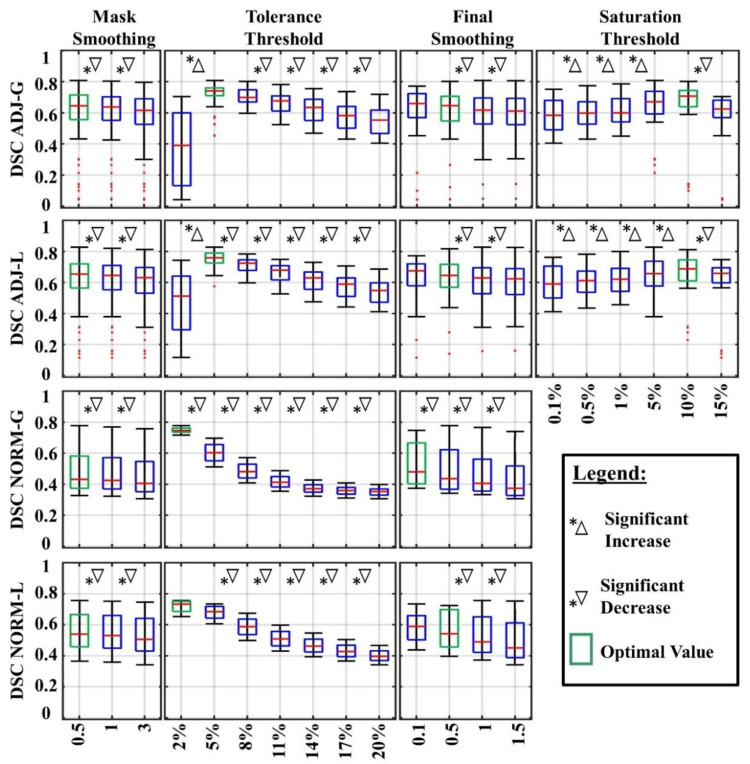
DSC changes using different parameter values. Each boxplot shows the DSC of all the pipelines where one parameter was set to a certain value. Significant increases and decreases are indicated with a star and a triangle. Boxbplots corresponding to the optimal values are in green. Top edges of the boxes indicate the 25th and 75th percentiles.

By varying all the parameter settings, we tested 504 different segmentation pipelines for each ADJ method and 84 for each NORM method. Figure 4 shows for each standardization method (ADJ-L, ADJ-G, NORM-L, NORM-G) how the DSC changed when using different values of mask smoothing, tolerance threshold, final smoothing and saturation threshold. The values that significantly improved the DSC are highlighted with green boxes and summarized in Table 2, which reports the best-performing segmentation settings (optimal values) for each standardization method. When two values did not show significant difference in DSC across the set of tested pipelines, we identified the value giving the highest proportion of good DSC (>0.6) in our cohort, or for the final smoothing parameter, we opted for the smoothest output (see Figure 4).

Overall, we obtained excellent performance for 15 pipelines (7.9%) of the ADJ-L method (mean DSC across our cohort > 0.8) and 5 pipelines (4.2%) for the ADJ-G method, while when using the NORM-L or NORM-G methods, no pipeline showed a median DSC above 0.80.

**Figure 5:**
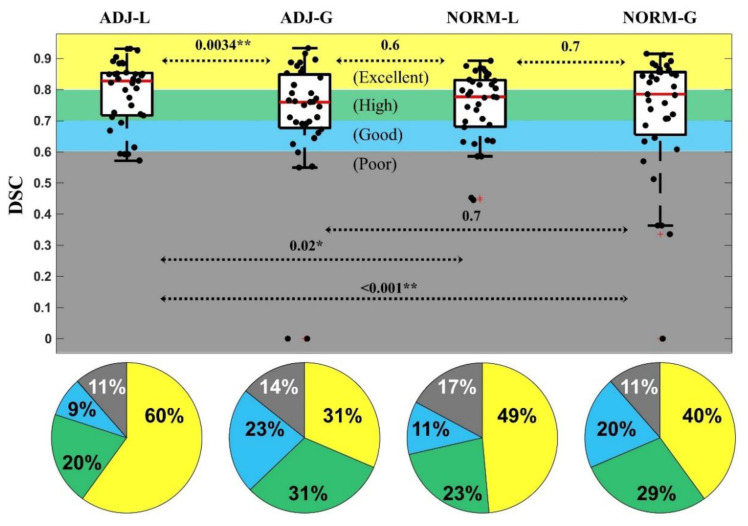
DSC for the best-performing pipeline of each standardization method. Pies indicate the percentage of patients with excellent similarity (yellow, DSC > 0.8%), high similarity (green, DSC from 0.7 to 0.8), good similarity (blue, DSC from 0.6 to 0.7), and poor similarity (DSC < 0.6). Significant differences (Wilcoxon signed-rank test) between the standardization methods are indicated with asterisks (* *p* < 0.05; ** *p* < 0.01). Data points beyond the boxplot whiskers are indicated with +.

Figure 5 shows the results when looking at the optimal pipeline for each standardization method (obtained using the values indicated in Table 1): we obtained excellent DSC in 60% of our patients with ADJ-L, 49% with NORM-L, 40% with NORM-G and 31% with ADJ-G (see Figure 5). In addition, the ADJ-L segmentation pipeline presented higher DSC than ADJ-G (*p* = 0.0034), NORM-G (*p* < 0.001) and NORM-L (*p* = 0.02 marginally significant) as shown in Figure 5. Based on these findings, all the following analyses were conducted on the optimized performing pipeline for the ADJ-L.

### 3.1. Inter-Rater Analysis

The segmentation outputs we obtained when varying the initial seed were identical in almost all cases. In the first scenario (two different seeds selected from same view), we had excellent DSC (axial and sagittal: 1 (1–1); coronal: 1 (0.996–1)) and volume ratios (axial and sagittal: 1 (1–1); coronal: 1 (0.992–1)) in our cohort. In the second scenario (two different seeds selected from different views), we also found excellent DSC of 1 (0.999–1) and high volume ratios of 1 (0.998–1).

### 3.2. Anatomical Report

**Figure 6:**
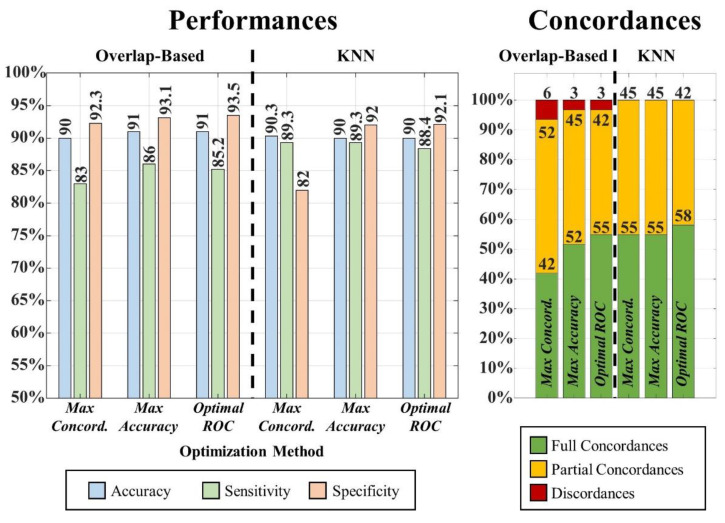
Anatomical report results. Overall performances (left bar plot) and individual reports’ concordance (right bar plot) for overlap-based (left) and KNN-label-substitution (right) approaches.

We generated an automated anatomical report of the resection output for each patient using the different approaches described in Section 2.5 and obtained the performance showed in Figure 6. The LOOCV used to define the optimal threshold on the resection percentage (to classify an area as “resected”) gave very similar threshold values (1.76–1.77%) when using the three different optimization methods, although the performance varied depending on them (as reported in Figure 6).

By comparing the overlap-based and the KNN-based approach, we observed that the KNN outperformed the overlap-based approach in terms of the overall number of automated reports that were concordant with the gold standard: Figure 6 (left panel) shows how the KNN approach did not produce any discordant report independently from the LOOCV method used to define the threshold on the resection percentage; contrarily, the overlap-based approach generated a discordant report in one (3%) or two cases (6%). Moreover, Figure 6 also reports the results in terms of overall accuracy, sensitivity and specificity of the automated output (resected versus non-resected anatomical areas) across the entire cohort, showing how both approaches present very high performance (accuracy: 90–91%; sensitivity: 85–89%; specificity: 82–94%). Based on the highest number of fully concordant automated reports, we identified the KNN approach, which used the threshold computed via optimal ROC curve point, as the best-performing method for anatomical-report generation; thus, this method was implemented in the GUI presented in this study.

### 3.3. Graphic User Interface (GUI)

**Figure 7:**
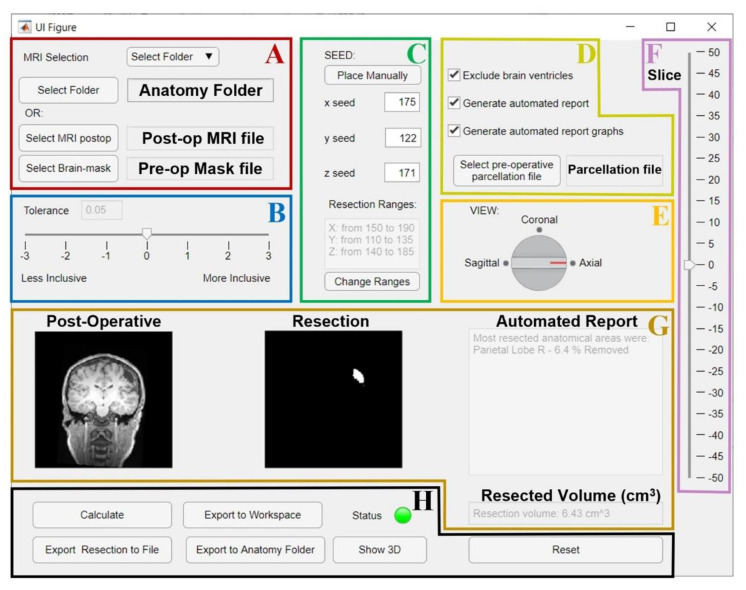
GUI for the resection segmentation. (**A**): Interface to select postoperative MRI and preoperative brain-mask files, or the patient’s Brainstorm anatomy folder that contains them. (**B**): Slider to select tolerance threshold (default: optimal value). (**C**): Interface to place the initial seed using an MRI viewer (see Figure 8) or entering its coordinates. The user can set an additional ROI for region growing by entering its ranges (this is optional, see Figure 8). (**D**): Options to exclude brain ventricles and/or generate the anatomical report through a brain parcellation file, which must be selected. E and F: the user can change the view (**E**) and slice (**F**) to visualize the postoperative MRI and resection model shown in G. (**G**): Preview of results (left: postoperative MRI; middle: resection model, right: automated report and resection volume in cm^3^). (**H**): Buttons to run the segmentation (“Calculate”), or to export results to a file (NIfTI), MATLAB workspace, or directly to the initial Brainstorm anatomy folder. Status LED turns red in case of error (green otherwise). “Show 3D” button shows the resection model (see Figure 8), while “Reset” allows to reset all settings and run again.

**Figure 8:**
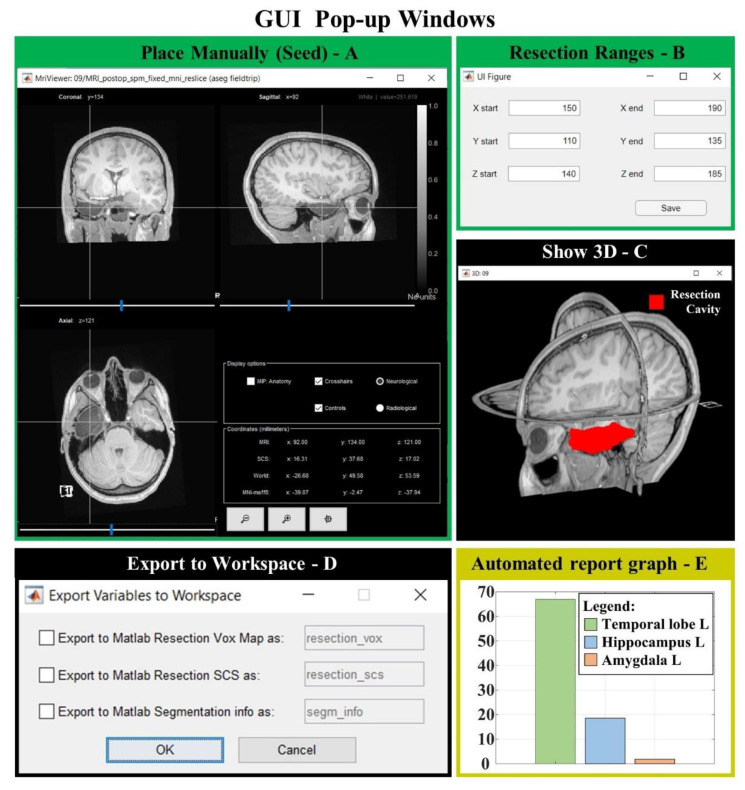
GUI popup windows. (**A**) MRI viewer for seed placement (when “Place Manually” is clicked). (**B**) Window for the definition of an optional ROI in which the region is free to grow (when “Change Ranges” button is clicked). (**C**) 3D MRI viewer from Brainstorm software (if “Show 3D” button is clicked) shows the 3D resection output (red). (**D**) If “Export to Workspace” is clicked, the user can export the output in voxel coordinates and/or in Subject Coordinates System (SCS), as well as the segmentation parameters and seed coordinates (Segmentation info). (**E**) Bar graph of the anatomical report (when “Generate automated report graphs” is selected).

The best-performing segmentation pipeline and anatomical-report approach were finally integrated in a GUI, which is outlined and described in Figure 7 and Figure 8, for a user-friendly creation of the resection model. Given a preoperative brain mask and a postoperative MRI, the GUI produces a 3D segmentation model of the resection with minimum user intervention (that is needed for the seed placement only). The presented GUI is a Matlab application, freely available on GitHub (https://github.com/rbillardello/BrainResectionApp (accessed on 14 April 2022)).

To use the GUI, the user can manually select the inputs (i.e., the coregistered postoperative MRI and preoperative brain-mask files) or, alternatively, the patient’s anatomy folder of the Brainstorm software database containing them. As a preoperative brain mask, the GUI accepts the mask generated through Freesurfer (the results reported above were obtained using this type of input), but also through Brainstorm using SPM (http://www.fil.ion.ucl.ac.uk/spm/ (accessed on 10 January 2020)) or Fieldtrip [45]: in these last two cases, the definition of the mask for the region-growing algorithm is obtained by excluding all the voxels labeled as CSF fluid, skull, scalp or background.

Once the postoperative MRI is selected, the user can manually place the initial seed on the Brainstorm MRI viewer (see Figure 7C and Figure 8A), which can be directly opened from our GUI (previous installation of the Brainstorm MATALB Toolbox is required). The GUI then provides the user a preview of the result (see Figure 7G) that helps tune the tolerance threshold that is set to the default optimal value, but can be modified by the user (Figure 7B); all the other parameters instead are fixed to their optimal values.

**Figure 9:**
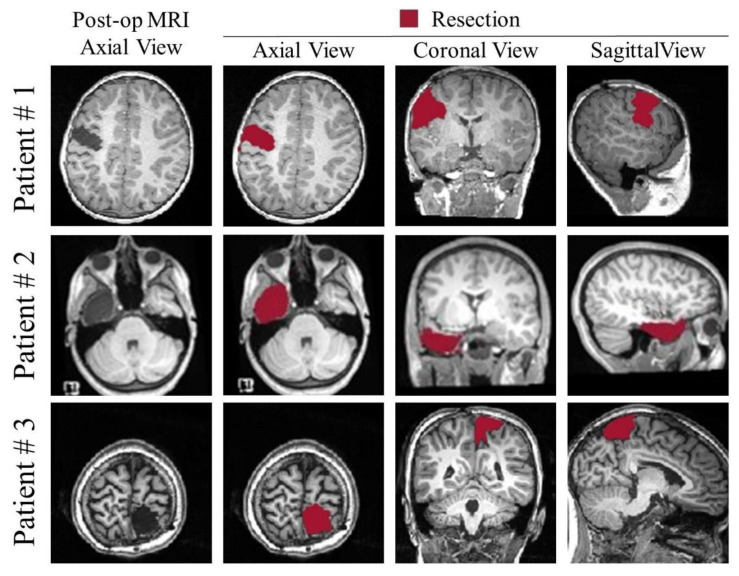
Examples of segmentation from the presented GUI from three different patients: left frontal resection (#1), left temporal resection (#2); right parietal resection (#3).

Finally, by using a preoperative cortical-surface-parcellation file, the GUI can also provide an automated anatomical report of the resection, as previously described. The segmentation output can be exported to the MATLAB workspace or as NIfTI file or directly saved into the patient’s anatomy folder in the Brainstorm software database (if this was selected as input to the GUI). Figure 9 shows three examples of the resection model from three of our patients.

### 3.4. Single-Parameter Tuning

We estimated that the highest DSC that can be obtained through the GUI by varying only the tolerance threshold (MaxS) was 0.84 (0.77–0.88). Compared with the maximum possible values obtainable by modifying all the parameters of our algorithm, we estimated an MQD of 0.05 (MaxP = 0.86 (0.82–0.91)). Such a low MQD is desirable, as it implies that the user can reach by only varying one single parameter (from seven possible choices)—a performance that is very similar to that of a user who can vary all of them (504 possible combinations of choices).

Furthermore, our data show that the obtained performance (MaxS) for all standardization methods did not depend on the patient’s age (ADJ-G: *p* = 0.58, R = 0.098; ADJ-L: *p* = 0.48, R = 0.12; NORM-G: *p* = 0.65, R = 0.079; NORM-L: *p* = 0.82, R =−0.040) or the resection volume (ADJ-G: *p* = 0.61, R = 0.090; ADJ-L: *p* = 0.77, R = 0.051; NORM-G: *p* = 0.67, R = 0.075; NORM-L: *p* = 0.38, R = −0.15); neither did they differ between temporal and extratemporal cases (ADJ-G: *p* = 0.60; ADJ-L: *p* = 0.56; NORM-G: *p* = 0.51; NORM-L: *p* = 0.80).

## 4. Discussion

Neuroimaging and neurophysiology research of epilepsy biomarkers ask for accurate delineation of the resected brain region within the patient’s MRI. In drug-resistant epilepsy, the “epileptogenic zone” (EZ) within the brain can only be determined post-surgically: one can be certain that the EZ was correctly identified only after the surgery renders the patient seizure-free [6,46], as this is the only proof that the EZ was indeed excised. The resected brain tissue thus represents the best estimate of the EZ, and its accurate definition post-surgery is critical to all the studies investigating and validating novel methods to estimate the EZ or predict outcomes in drug-resistant epilepsy.

For these reasons, there is an evident need for a user-friendly tool that is specifically designed to accurately delineate resection cavities and does not require fine tuning of a variety of image-processing parameters, which is not always intuitive and approachable to a nontechnical user. Through this study, we aimed at providing the epilepsy-research community with a user-friendly validated tool for this purpose. Although MRI segmentation tools are available, we present here a new user-friendly application for the epilepsy-research community, which to our best knowledge is unique in the following aspects:

(i) It is specifically tailored and tuned for a quick and reliable definition of epilepsy surgical resections in the MRI space by any user that lacks advanced expertise in medical image segmentation;
(ii) It provides an automated anatomical report of the resected brain tissue at the lobar and sublobar level (when integrated with the use of other segmentation software).

We introduced and validated a semiautomated segmentation pipeline consisting of a hybrid approach that integrates a traditional region-growing method on the postoperative MRI with the patient-specific preoperative brain mask: our validation results in a cohort of 35 patients show how we could optimize the segmentation pipeline and obtain excellent levels of accuracy to the hand-drawn gold standard (mean dice coefficient of 83%) independently from the initial seed placement (which was left as the main user-dependent input).

In addition, this study presents the first segmentation tool, to our best knowledge, designed and validated to automatically outline an anatomical report of the surgical excision on the MRI, and we report accuracies above 90% to the clinical MRI reports. We tested different methods to generate an anatomical report (see Figure 6): the better performance of the KNN approach (which assigns all the resected voxels to the closest anatomical area based on the preoperative cortical parcellation file), compared to the overlap-based approach, suggests that can minimize the effect of slight MRI co-registration errors (between pre- and postoperative MRIs). Thus, we implemented such an approach in the publicly available GUI.

We performed validation in the context of 35 pediatric epilepsy surgery cases, which include an heterogenicity of anatomical regions (differently from adult epilepsy cases who are predominantly temporal lobe epilepsy). Our findings showed that the performance of our proposed segmentation did not depend on the patient’s age (ranging in our cohort from 2 to 21 years), the resection volume or on the type of surgery (whether temporal or extratemporal cases). Therefore, the excellent performance that we report suggests reliability of the proposed application to a variety of clinical scenarios and provides evidence for adopting it as the segmentation solution for neuroimaging research in epilepsy surgery.

We finally presented and made publicly available an open-source, user-friendly application for medical image processing that can be used by the epilepsy research community to extract the volume and margins of a resection cavity in 3D MRIs along with its neuroanatomical labeling (anatomical resection report). The GUI is focused entirely and specifically on segmenting the resection cavity and it was designed to maximize user efficiency and the output accuracy, as:

(i) we identified the optimal values for a variety of image-processing parameters so that no tuning is needed (which would be otherwise difficult for anyone without expertise in image processing);
(ii) we left the simplified parameter selection and kept it to a minimum: we only left the user the option to vary one parameter and made its tuning through the GUI intuitive (see Figure 7B);
(iii) we demonstrated how by varying one single parameter (among seven possible choices), the user can reach a performance that is very similar to that of a user who can vary all of them (504 choices)
(iv) the user is only asked to place an initial seed anywhere in the resection cavity that they desire to delineate (Figure 7C and Figure 8A).

### Limitations and Future Directions

Validation on a larger dataset would be beneficial; however, taking into account the incidence rate of epilepsy surgery, our sample size (*n* = 35) can be regarded as satisfactory. Since the source code is freely available, reproducibility and future validation on different populations is encouraged and warranted to further ensure generalizability of our results.

The proposed segmentation pipeline cannot be used in case of laser ablation, which is becoming a more and more common surgical option for children with DRE. Further development and validation are needed to allow delineation of the ablated brain tissue as their MRI characteristics largely differ from brain resections.

## 5. Conclusions

Although delineation of the resected brain cavity on the MRI of patients who had epilepsy surgery is key for identifying biomarkers of the EZ, the current gold standard remains manual slice-by-slice tracing by experts. Here, we presented a novel user-friendly open-source GUI that implements a semiautomated segmentation pipeline specifically optimized to generate resection models and their anatomical reports from patients who had epilepsy neurosurgery, while minimizing the user interaction. The GUI was validated on a cohort of 35 patients, demonstrating excellent performance, and was made publicly available to the research community.

## Figures and Tables

**Table 1:** Parameters and tested values.

Parameter	Applied to	Tested Values	Standardization	
			ADJ-G ADJ-L	NORM-G NORM-L
**Smoothing**	Brain Mask	0.5; 1; 3	3 Values	
**Tolerance Threshold**	Region-Growing	2%; 5%; 8%; 11%; 14%; 17%; 20%	7 Values	
**Saturation Threshold**	Post-op MRI	0.1%; 0.5%; 1%; 5%; 10%; 15%	6 Values	n/a
**Smoothing**	Output	0.1; 0.5; 1; 1.5	4 Values	
**Total Number of Combinations (Segmentation Pipelines)**			504	84

Smoothing: applied to the brain mask or final resection output, it is the standard deviation of the Gaussian filter for smoothing; Tolerance Threshold: applied to region-growing algorithm, it is the maximum difference that a voxel can have from the average of the growing region to be included in it; Saturation Threshold: applied to postoperative MRI, contrast adjustment saturates bottom and top percentage of all pixels.

**Table 2:** Optimal parameter values and their associated DSC.

Parameter	ADJ-G	ADJ-L	NORM-G	NORM-L
**Mask Smoothing**	0.5	0.5	0.5	0.5
**Tolerance Threshold**	0.05	0.05	0.02	0.02
**Final Smoothing**	0.5	0.5	0.1	0.5
**Saturation Threshold**	10%	10%	NA	NA
**DSC Median (IQR)**	0.76 (0.68–0.85)	0.83 (0.72–0.85)	0.78 (0.68–0.83)	0.79 (0.66–0.86)

## Data Availability

The data presented in this study are available on request to the corresponding author. The data are not publicly available due to privacy restrictions. The GUI source code is freely available as a MATLAB-based application (https://github.com/rbillardello/BrainResectionApp (accessed on 14 April 2022)).
